# Evidence for a memory advantage for prosocial behaviors

**DOI:** 10.1002/brb3.3096

**Published:** 2023-06-08

**Authors:** Pauline Urban Levy, Allison M. Sklenar, Andrea N. Frankenstein, Eric D. Leshikar

**Affiliations:** ^1^ Department of Psychology University of Illinois at Chicago Chicago, Illinois USA

**Keywords:** impression formation, impression memory, memory, prosocial behavior, recognition

## Abstract

**Introduction:**

Prior work in the memory domain has shown that certain social information is especially well‐remembered such as information for social targets who cheat. Less work, however, has investigated the extent people remember information for social targets who engage in prosocial behaviors (e.g., helping) in social interactions. The current investigation examines whether there is a memory advantage for social targets who perform prosocial behaviors.

**Methods:**

Across two experiments, participants formed impressions of social targets engaging in prosocial and non‐prosocial behaviors. Participants were then tested on their memory for the impression as well as the specific behavior each social target performed.

**Results:**

Results of Experiment 1 showed that memory for impressions was better for social targets engaging in prosocial compared to non‐prosocial behaviors. Results of Experiment 2 showed marginally better behavior memory for targets performing prosocial compared to non‐prosocial behaviors.

**Conclusion:**

Overall, results of both experiments provide converging evidence of a prosocial advantage in memory, which suggests that people are attuned to prosocial behaviors exhibited by others in the social domain.

## Graphical abstract

Given the importance of prosociality in social contexts, the current investigation examines whether people show a memory advantage for social targets who perform prosocial behaviors. Results of both experiments showed evidence of a prosocial advantage in memory, which has not been shown before. Overall, results suggest that people are attuned to prosocial behaviors exhibited by others.

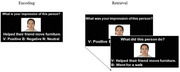

## 1 A MEMORY ADVANTAGE FOR PROSOCIAL BEHAVIORS

Prosocial behaviors are those that benefit another person other than the self (Batson & Powell, 2003). Past work has investigated the importance of prosocial behaviors in the social domain, such as understanding motivations to engage in prosocial activities (Barry & Wentzel, 2006; Eisenberg & Fabes, 1990; Mayr & Freund, 2020; Piff & Robinson, 2017; Weinstein & Ryan, 2010). Other work demonstrating the importance of prosocial behaviors has shown that expression of prosocial behaviors (e.g., performing prosocial behaviors) develops early in childhood (Malti & Dys, 2018), and further, neuroimaging evidence suggests that prosocial behaviors induce a strong neural response in social and reward processing systems of the brain (Morelli et al., 2018a). Although this past work has demonstrated the importance of prosocial behaviors, less work has investigated the extent that information that is prosocial in nature might be especially well‐remembered, and thus exhibits a prosocial advantage in memory. In this investigation, we investigate whether episodic memory (e.g., memory for specific details associated with specific social targets) is better for targets who engage in prosocial versus non‐prosocial behaviors.

Work on person memory (i.e., memory representations for previously encountered social targets) suggests that some types of behaviors tend to be especially well‐remembered. For example, some work shows that memory for unfair or cheating behaviors are typically well‐remembered compared to control behaviors (Bell & Buchner, 2009, 2012; Bell et al., 2012a, 2012b, 2010; Buchner et al., 2009; Schaper et al., 2019). In one investigation, participants were exposed to social targets who showed cheating behaviors, and other targets who showed non‐cheating behaviors. Results indicated that participants had better memory for targets who engaged in cheating behaviors, suggesting an advantage in memory for cheaters (Bell & Buchner, 2012). Work in so‐called cheater detection studies suggest the improved memory for cheating behavior may be useful in guiding future interactions with targets (i.e., avoid those who cheat). Importantly, it may be that other types of behaviors, such as prosocial behaviors, show similar advantages in memory. Given the importance of prosocial behaviors in the social domain, it may be that prosocial behaviors are also memorable because they may be used to guide future behaviors (i.e., approach those who help). Although less work has been devoted to understanding memory for targets who engage in prosocial behaviors, there is some work that hints that people are attentive to targets who perform prosocial activities. For example, in one investigation (Morelli et al., 2018b), college students were prompted to identify peers who were “socially valuable” in their dormitory, which importantly included peers who engaged in prosocial behaviors. In a second part of that experiment, participants viewed images of the social targets (i.e., peers) identified as engaging in prosocial behaviors while their brains were scanned using fMRI. Results indicated that participants showed heightened brain activity in the medial prefrontal cortex (an area known to be involved in social cognitive processing; Mitchell et al., 2005) for targets showing prosocial behaviors compared to other targets who did not engage in such behaviors. Although this study was not a memory investigation per se, these findings imply that people may be especially prone to attend to social targets who perform prosocial behaviors in their social network, which could underlie improved memory for targets exhibiting such behaviors.

Theoretical work in person memory suggests there are different types of details about social targets that are represented in memory (Srull & Wyer, 1989). This past work suggests that people can remember detailed episodic information (e.g., specific behaviors that targets engage in), but also remember more abstracted, or gist‐like, information such as whether one has an overall positive or negative impression of a target. One way to measure memory for both specific behaviors and impressions is with impression memory experimental procedures. In impression memory procedures, participants are shown targets engaging in different behaviors and are asked to form positive, negative, or neutral impressions of targets (Cassidy et al., 2013; Todorov & Uleman, 2003). Participants then complete a memory test where they are asked to report whether they formed a positive, negative, or neutral impression of the targets they saw before, and then further, they are asked to report which behavior the target performed. Such an experimental approach allows assessment of memory for both the impression generated about the target, as well as the specific behavior each target performed. Interestingly, some past work has shown that there can be different patterns of memory for impressions versus behaviors (Leshikar et al., 2015b; Leshikar & Gutchess, 2015a; Todorov & Uleman, 2003). For example, although not an investigation of prosocial behaviors, one study found improved impression memory for targets who were similar to the self (e.g., a type of self‐reference effect for social targets), but behavior memory for those same exact targets did not show a similar memory effect (Leshikar & Gutchess, 2015b). Because impression versus behavior memory can differ even for the same exact social target, it can be advantageous to assess memory for both impressions and behaviors to gain a more complete understanding of how a variety of details about targets are remembered. Thus, in this investigation, we examine memory for impressions and behaviors for targets who engage in prosocial versus non‐prosocial behaviors to better understand how prosocial information is represented in memory.

Across two experiments, we examine both impression and behavior memory for targets associated with prosocial relative to non‐prosocial behaviors to investigate potential memory advantages for prosocial information. In both experiments, participants formed positive, negative, or neutral impressions of targets based on behaviors, before then completing a memory test assessing both impression and behavior memory. We make two predictions in this investigation. First, for impression memory, we predict that memory will be better for targets associated with prosocial relative to non‐prosocial behaviors. Such a finding would add more evidence that some types of behaviors are more memorable than others. Second, for behavior memory, we predict that memory for prosocial behaviors will be better than non‐prosocial behaviors. Observing evidence in line with both predictions would provide complementary evidence in favor of a prosocial advantage in memory, and further, be aligned with past work suggesting the importance of prosociality in the social domain (Barry & Wentzel, 2006; Eisenberg & Fabes, 1990; Malti & Dys, 2018; Mayr & Freund, 2020; Morelli et al., 2018a, 2018b; Piff & Robinson, 2017; Weinstein & Ryan, 2010). Overall, results of this investigation will add to the body of work showing how memory for details associated with social targets is represented in memory.

## 2 EXPERIMENT 1

### 2.1 Methods

#### 2.1.1 Participants

Fifty‐five undergraduates (32 female; ages 18–24, *M* = 19.51, *SD* = 2.02) were recruited to participate in this experiment from the University of Illinois at Chicago. A power analysis conducted in G*Power (Faul et al., 2007) for a one‐way repeated measures ANOVA showed that we would need 39 participants to detect a smaller effect size of *d* = .3. Given the novelty of this research question, we chose a more conservative effect size for this power analysis. Two participants were removed from analysis due to incomplete data. Consent was obtained from each participant in accordance with the Institutional Review Board guidelines. Participants received course credit for their participation in the study.

#### 2.1.2 Stimuli

We used 96 faces and 96 behavioral sentences in this experiment. Faces were taken from the Chicago Faces Database (Ma et al., 2015) and included an equal number of male and female faces as well as an equal number of targets from the following groups: Black, Asian, Latinx, and White. All faces exhibited a neutral expression. There were three different types of behavioral sentences: 32 *prosocial individual* behaviors, 32 *prosocial group* behaviors, and 32 *non‐prosocial* behaviors. *Prosocial individual* sentences described behaviors prosocial in nature which benefitted a single individual (e.g., helped someone change their car tire). *Prosocial group* sentences described prosocial behaviors that benefitted multiple people (e.g., brought snacks to a group meeting). *Non‐prosocial* sentences described behaviors that did not benefit another person (e.g., walked home after a long day at work). We included two types of prosocial behaviors to explore how different kinds of prosocial behaviors might affect memory.^1^ All sentences ranged from 3 to 12 words in length across the three conditions (*M* = 6.26, *SD* = 1.94). Behaviors for the prosocial individual and prosocial group sentences were adapted from prior literature (Amato, 1990; Brown et al., 2008, 2003; Doeglas et al., 1994; Jones & Perlman, 1987; Maisel & Gable, 2009; Neff & Karney, 2005; Philippe Rushton et al., 1981) as well as created by the experimenters. Behavior sentences were normed for valence and prosociality prior to the current study (See Supporting Information). Across participants, face and behavior stimuli were counterbalanced to appear in the encoding phase or as novel (lure) items at retrieval.

#### 2.1.3 Procedure

**FIGURE 1:**
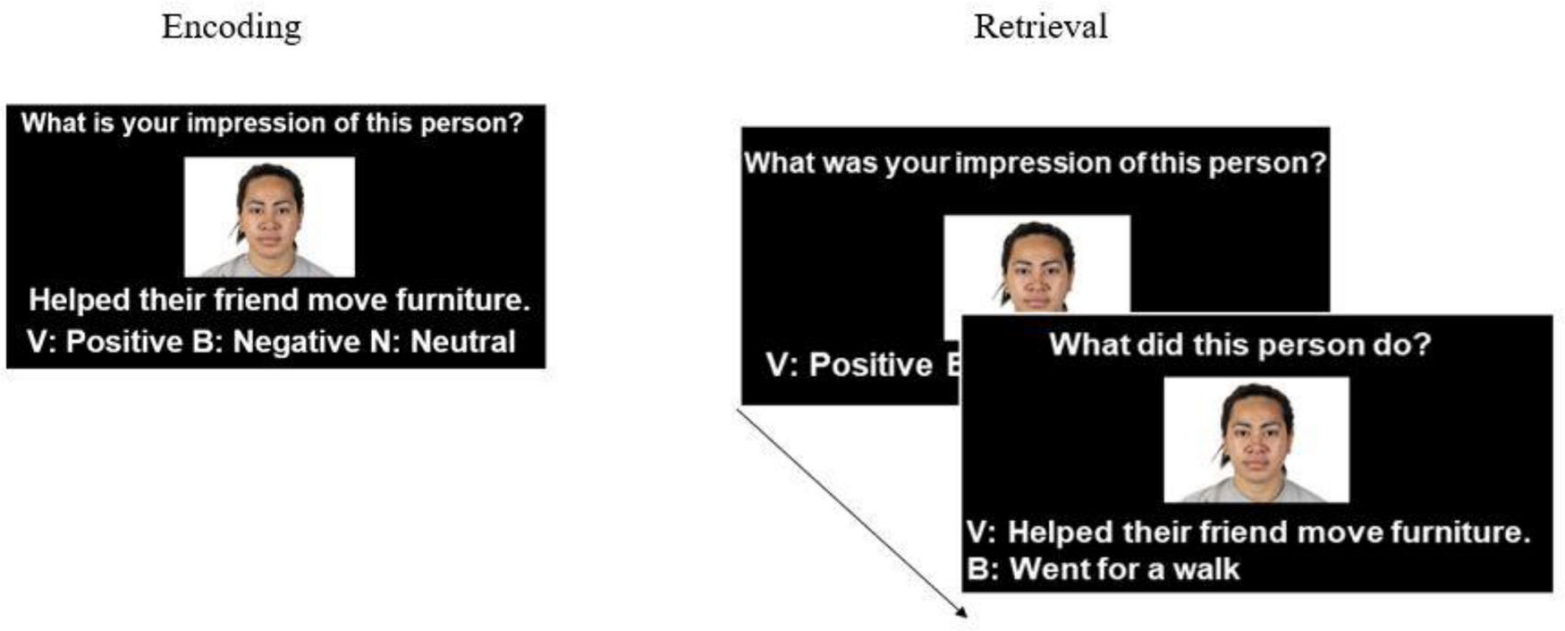
Trial schematic for both the encoding (i.e., impression formation) and retrieval phases of Experiments 1 and 2.

After giving informed consent, participants sat at a computer in an individual cubicle in a quiet room and were prompted to begin the experiment. The experiment consisted of two phases: encoding (i.e., impression formation) and retrieval. Before starting the experiment, participants were first given training on the encoding task that included two practice trials. Participants were encouraged to ask any questions or clarifications about the procedure. After training, participants completed the encoding phase. During the encoding phase, participants formed impressions for 72 targets (24 prosocial individuals, 24 prosocial group, 24 non‐prosocial). On each encoding trial, participants were shown a face and a behavior (either prosocial group, prosocial individual, or non‐prosocial) and were asked, “What is your impression of this person?” Participants were instructed to form either a positive, negative, or neutral impression for each target based on the targets face as well as on the behavior. Participants indicated their impression with a keyboard keypress (v = positive | b = negative | n = neutral; see Figure 1). Encoding phase trials were self‐paced. Face‐behavior pairs were presented in a random order.

Immediately following encoding (without a delay), participants then completed a short training on the retrieval phase task which included two practice trials. After completing the training, participants were given an opportunity to ask questions about the task. The retrieval phase consisted of 96 trials, which included 72 studied “old” targets seen during encoding as well as 24 novel targets not seen before. We measured memory for impressions (i.e., the impression the participants formed about targets) and behaviors associated with targets. On each retrieval phase trial, participants were shown a target face and asked, “What was your impression of this person?” Participants reported whether they formed a positive, negative, or neutral impression of the target, or whether the target was a novel target they had not seen before (v = positive | b = negative | n = neutral | m = new). This served as our impression memory decision. Then, while the same face was still on screen (same face as the impression memory decision), participants were then asked, “What did this person do?” and were shown two behaviors along with an option to say that the face was novel (e.g., v = Went to the park | b = Held the door open for someone | m = New). For old trials, one behavior was the correct behavior the target performed and the incorrect lure was a behavior previously seen, but associated with a different target. Incorrect lures could be from the same condition as the correct behavior (e.g. if the correct behavior was a prosocial group behavior, the lure could be from the prosocial group behavior as well) or from a different condition (e.g. if the correct behavior was a prosocial group behavior, the lure could be from the non‐prosocial category, etc.). Retrieval phase decisions were self‐paced. Retrieval trials were presented in a randomized order. After participants completed retrieval, they were debriefed and dismissed. Overall, participants took about 35 min to complete the full experimental procedures.

### 2.2 Results

In this section, we report results of the encoding (i.e., impression formation) and retrieval phases of this experiment. For encoding (impression formation), we report the percent of all positive, negative, and neutral impression trials across our three conditions (prosocial individual, prosocial group, non‐prosocial), respectively. Specifically, we took all the trials that were given a positive impression, and examined the percent of those positive impression trials that were prosocial individual, prosocial group, and non‐prosocial (and we used an analogous approach for the negative and impression trials). Results of this analysis showed that participants formed positive impressions significantly more often for prosocial individual (*M* = .41, *SD* = .07), *t*(104) = 13.85, *p* < .001, *d* = 3.17, and prosocial group behaviors (*M* = .45, *SD* = .07), *t*(104) = 15.73, *p* < .001, *d =* 3.55, compared to non‐prosocial behaviors (*M* = .14, *SD* = .10). Further, participants formed significantly more neutral impressions for non‐prosocial behaviors (*M* = .66, *SD* = .24) compared to prosocial individual (*M* = .18, *SD* = .15), *t*(102) = −11.046, *p* < .001, *d* = −2.44, and prosocial group behaviors (*M* = .16, *SD* = .13), *t*(102) = −11.64, *p* < .001, *d* = −2.7. Participants also formed significantly more negative impressions for non‐prosocial (*M* = .61, *SD* = .3) behaviors than they did for the two prosocial conditions (prosocial individual: (*M* = .20, *SD* = .18), *t*(74) = −6.40, *p* < .001, *d* = −1.76; prosocial group: (*M* = .19, *SD* = .21), *t*(74) = −6.45, *p* < .001, *d* = −1.67).^2^

For retrieval, we report both impression memory (memory for the self‐generated impressions associated with targets) and behavior memory (memory for the behaviors associated with targets; See Tables 1 and 2). First, we examined how well participants were able to retrieve the impressions they formed about targets during the encoding phase of the experiment. We calculated impression memory as the percent of trials where the impression generated at encoding was correctly identified, out of the times they made an impression memory response for previously seen targets (correct impression/[correct impression + incorrect impression]), as done before (Bayen et al., 1996; Giannakopoulos et al., 2021; Leshikar et al., 2015b, 2016; Leshikar & Gutchess, 2015a, 2015b; McCurdy et al., 2017, 2019, 2020a, 2021; Murnane & Bayen, 1996). We entered impression memory into a repeated‐measures ANOVA split by behavior type (prosocial individual, prosocial group, non‐prosocial) and found a significant difference between the three groups *F*(1.68, 87.36) = 15.06, *p* < .001. Results indicated that participants remembered their impressions of prosocial individual (*M* = .65, *SD* = .20), *t*(104) = 4.91, *p* < .001, *d =* .85, and prosocial group (*M* = .64, *SD* = .21), targets significantly more often than non‐prosocial targets (*M* = .48, *SD* = .21), *t*(104) = 4.58, *p* < .001, *d =* .79, suggesting a memory advantage for prosocial compared to non‐prosocial behaviors. There was no difference, however, between the two prosocial groups, *t*(104) = 0.33, *p* = .941.

**TABLE 1:** Mean proportions of correctly remembered prosocial individual, prosocial group, and non‐prosocial impressions in Experiments 1 and 2.

		Impression memory accuracy		
		Correct	Incorrect	New
Experiment 1. Items as a function of:				
	Behavior type			
	Prosocial individual	0.44	0.24	0.32
	Prosocial group Non‐prosocial	0.44 0.33	0.25 0.35	0.31 0.32
Experiment 2. Items as a function of:				
	Behavior type			
	Prosocial	0.44	0.28	0.28
	Non‐prosocial	0.44	0.28	0.28

*Note*: “New” refers to trials where previously seen (i.e., “old”) targets were incorrectly identified as new targets.

**TABLE 2:** Mean proportions of correctly remembered prosocial individual, prosocial group, and non‐prosocial behaviors paired with targets at encoding in Experiments 1 and 2.

		Behavior memory accuracy		
		Correct	Incorrect	New
Experiment 1. Items as a function of:				
	Behavior type			
	Prosocial individual	0.48	0.16	0.36
	Prosocial group Non‐prosocial	0.48 0.48	0.17 0.17	0.35 0.35
Experiment 2. Items as a function of:				
	Behavior type			
	Prosocial	0.55	0.13	0.31
	Non‐prosocial	0.52	0.16	0.32

*Note*: “New” refers to trials where previously seen (i.e., “old”) targets were incorrectly identified as new targets.

Turning to behavior memory, we calculated the percent of trials where the behavior shown during encoding was correctly recognized. We analyzed behavior memory using a repeated‐measures ANOVA split by behavior type (prosocial individual, prosocial group, and non‐prosocial). Results showed that behavior memory did not differ across the three types of behavioral sentences: prosocial individual (*M* = .48, *SD* = .16), prosocial group (*M* = .48, *SD* = .17), and non‐prosocial (*M* = .48, *SD* = .20), *F*(1.87, 97.09) = 0.03, *p* = .959, *d* = .13.

### 2.3 Discussion

In Experiment 1, participants remembered their impressions of social targets that displayed prosocial behaviors significantly more often than those that displayed non‐prosocial behaviors. For behavior memory, there was no difference in performance between prosocial and non‐prosocial behaviors. Overall, the findings from Experiment 1 showed evidence of a memory advantage for impressions of prosocial behaviors, consistent with our predictions. Experiment 1 also demonstrates a possible difference between impression memory and behavior memory, which is aligned with past work showing differences in memory for impressions versus behaviors suggesting that more gist‐like representations (like impressions) and more specific representations (like behaviors) may be stored in memory somewhat differently (Leshikar et al., 2015b; Leshikar & Gutchess, 2015a, 2015b; Todorov & Uleman, 2003). One limitation of Experiment 1 was that there were more prosocial behaviors associated with targets compared to non‐prosocial behaviors at encoding (e.g., 48 targets associated with prosocial behaviors compared to 24 targets associated with non‐prosocial behaviors), which could have influenced memory for prosocial versus non‐prosocial stimuli. To further investigate potential prosocial advantages in memory, we conducted an additional investigation where we showed an equal number of targets associated with prosocial as well as non‐prosocial behaviors. Because we did not find memory differences between the two different categories of prosocial behaviors in Experiment 1 (prosocial individual and prosocial group), we only included a single type of prosocial behaviors in Experiment 2 (e.g., prosocial individual).

## 3 EXPERIMENT 2

### 3.1 Methods

#### 3.1.1 Participants

Fifty‐two (41 female; ages 18–23, *M* = 18.80, *SD* = 1.21) students from the University of Illinois at Chicago participated. Three participants were removed from analysis due to incomplete data. Participants gave informed consent to participate in the experiment in accordance with the University of Illinois at Chicago Institutional Review Board's requirements.

#### 3.1.2 Stimuli

Stimuli were identical to that of Experiment 1 except that the prosocial group behavioral sentences were removed.

#### 3.1.3 Procedure

Procedure was identical to Experiment 1 with the exception that participants completed fewer trials due to the removal of the prosocial group stimuli (i.e., 48 trials at encoding [24 prosocial, 24 non‐prosocial]; 72 trials at retrieval [24 prosocial, 24 non‐prosocial, 24 novel trials]).

### 3.2 Results

In this section, we report data from the encoding and retrieval phases. As in Experiment 1, we report the percent of all positive, negative, and neutral impression trials across our three conditions (prosocial individual, prosocial group, non‐prosocial), respectively. Results of this analysis showed that participants made significantly more positive impressions for targets displaying prosocial behaviors (*M* = .70, *SD* = .15) compared to non‐prosocial behaviors (*M* = .30, *SD* = .15), *t*(48) = 9.55, *p* < .001, *d* = 2.73. Participants formed significantly more neutral impressions for non‐prosocial behaviors (*M* = .71, *SD* = .21) compared to prosocial behaviors (*M* = .29, *SD* = .21), *t*(46) = 6.91, *p* < .001, *d =* 2.01. Participants also formed significantly more negative impressions of non‐prosocial behaviors (*M* = .76, *SD* = .23) than prosocial behaviors (*M* = .24, *SD* = .23), *t*(39) = 7.07, *p* < .001, *d* = 2.24.^3^

For retrieval, we calculated impression and behavior memory as we did in Experiment 1. For impression memory, results showed no significant difference in memory for targets associated with prosocial (*M* = .61, *SD* = .22) or non‐prosocial behaviors (*M* = .61, *SD* = .21), *t*(48) = 0.04, *p* = .97. In contrast, for behavior memory, we found a marginal effect where memory for prosocial behaviors (*M* = .55, *SD* = .17) was better than for non‐prosocial behaviors (*M* = .52, *SD* = .16), *t*(48) = 1.966, *p* = .055.

### 3.3 Discussion

In Experiment 2, our purpose was to further investigate the extent that memory for prosocial behaviors might show an advantage in memory, while also controlling for the limitation of Experiment 1 (by having equal number of prosocial and non‐prosocial trials). Results showed marginally better memory for targets who showed prosocial compared to non‐prosocial behaviors as measured by behavior memory, which is in line with the idea that memory may be enhanced for targets who engage in prosocial behaviors. It is worth noting, however, that we did not replicate the impression memory result in Experiment 2 given that we did not find improved memory for the prosocial compared to non‐prosocial condition. Although these effects differed from the evidence of enhanced memory for impressions seen in Experiment 1, results of Experiment 2 provided converging evidence in support of a prosocial advantage in memory.

## 4 GENERAL DISCUSSION

In this set of experiments, we examined memory for social targets associated with prosocial versus non‐prosocial behaviors to understand the extent that prosocial information shows an advantage in memory. We have two primary findings in this set of experiments. First, we found better impression memory for social targets exhibiting prosocial compared to non‐prosocial behaviors in Experiment 1. Second, we observed marginally better behavior memory for prosocial compared to non‐prosocial behaviors in Experiment 2. Both findings suggest existence of a memory advantage for prosocial information, which is aligned with past work suggesting prosociality plays an important role in the social domain (Barry & Wentzel, 2006; Eisenberg & Fabes, 1990; Malti & Dys, 2018; Mayr & Freund, 2020; Morelli et al., 2018a, 2018b; Piff & Robinson, 2017; Weinstein & Ryan, 2010). Overall, these data extend understanding of how memory for targets is represented, especially those who exhibit prosocial behaviors.

In this investigation, we examined the extent that *impression* memory for social targets would be better remembered if the basis of those generated impressions was on prosocial behaviors. Past experimental work investigating impression memory has shown that some types of information associated with targets shows an advantage in memory relative to other types of information. For example, some work has shown that when targets exhibit characteristics that are similar to the self, impression memory for such targets tend to be well‐remembered compared to individuals who are less similar to the self (Leshikar et al., 2015b; Leshikar & Gutchess, 2015a, 2015b; Sklenar et al., in press). In the current investigation, we found evidence that impression memory for targets who exhibited prosocial behaviors was better relative to targets who showed non‐prosocial behaviors, at least as measured in Experiment 1. This finding is consistent with past work showing that impression memory is better for some types of information, as well as other work suggesting an important role for prosocial behaviors in the social domain. Interestingly, one possible reason we saw enhanced impression memory for the prosocial behaviors is because such behaviors may tap into a construct that is known to have a strong effect on impression formation in general, and that is the construct of warmth (e.g., a constellation of attributes that include friendliness, sincerity, trustworthiness, etc.). Decades of research on impression formation suggest that people are especially attentive to behaviors that imply a target is warm (Fiske et al., 2007). Indeed, recent work even suggests that people who exhibit prosocial behaviors are perceived as high in warmth (Kawamura et al., 2021). Thus, it may be that improved memory for impressions of targets associated with prosocial behaviors may partially be related to perceptions of warmth. Future work might further examine the extent that the prosocial advantage in memory may be related to the construct of warmth. Additionally, finding improved impression memory is further aligned with past work in other domains that suggests that self‐generated information (such as impressions) can be well‐remembered under a variety of conditions (Giannakopoulos et al., 2021; McCurdy & Leshikar, 2022; McCurdy et al., 2017, 2019, 2021; McCurdy et al., 2020a, 2020b) and can vary depending on type or variety of stimuli that is self‐generated.

In addition to finding improved impression memory for targets showing prosocial behaviors, we also observed marginally better *behavior* memory for targets showing prosocial versus non‐prosocial behaviors in Experiment 2. Past work has demonstrated that memory for certain types of behaviors, such as cheating, are better remembered than other types of information (Bell & Buchner, 2009; Bell et al., 2012a, 2012b, 2010; Buchner et al., 2009; Schaper et al., 2019). Thus, our behavior memory finding is aligned with this past work and demonstrates a similar advantage in memory for prosocial behaviors. Past theoretical work on memory advantages for information about targets (such as cheating behaviors) suggests that such memory effects may be adaptive because they can guide future actions (e.g., avoiding targets who cheat; Bell & Buchner, 2012). Interestingly, such theoretical accounts further argue that such memory effects are useful, or adaptive, only to the extent that people can remember the association between targets and some behavioral attribute or characteristic (e.g., remembering *this* person is associated with *that* attribute, such as cheating; Bell et al., 2012b; Buchner et al., 2009; Schaper et al., 2019). Turning back to the present findings, in the case of both our impression and behavior memory findings in Experiments 1 and 2, we found that participants were remembering the target along with episodic details associated with the target engaging in prosocial behaviors (either the behavior the target performed, or the impression generated about the target). Thus, it may be that our finding of a prosocial advantage in memory where participant remember specific details (impressions; behaviors) associated with targets, may be adaptive and help guide future goal‐directed behaviors in social contexts. Examining conditions under which memory might be improved is an important pursuit (Bjork & Benjamin, 2011; Burden et al., 2021; Frankenstein et al., 2022; Ilenikhena et al., 2021; Leach et al., 2019; Leshikar & Duarte, 2012, 2014; Leshikar et al., 2012, 2015a, 2017; Matzen et al., 2015), and the results of the current investigation contribute to that empirical goal.

In this set of experiments, we found an advantage in memory for prosocial versus non‐prosocial information that emerged somewhat differently across experiments. Specifically, in Experiment 1, we found better impression memory for targets exhibiting prosocial compared to non‐prosocial behaviors, whereas in Experiment 2, we found enhanced behavior memory for targets associated with prosocial information. To understand why results may have differed across experiments, it is useful to look at past work describing how information about targets is represented in memory. Past theoretical accounts of how information about people is organized in memory suggests there are different types of representations, such as less detailed memory representations like impressions (or even traits inferred about targets) as well as more detailed representations such as specific behaviors targets performed (Srull & Wyer, 1989). Importantly, empirical work has shown that memory effects may differ across these different types of memory representations (impression; behaviors) for the exact same targets, suggesting that different types of memory representations are stored somewhat independently in memory (Todorov & Uleman, 2003). As one example, in one investigation that did not examine prosocial behaviors, memory for impressions and behaviors was measured for targets who were either similar (or dissimilar) to the self (Leshikar & Gutchess, 2015b). Results showed improved impression memory for targets similar to the self; however, an analysis of behavior memory for the exact same targets showed that there were no differences in memory across targets. Given that memory effects for impression and behaviors may differ, it is then possible to understand why memory effects differed in Experiment 1 versus Experiment 2. Looking at the experimental procedures across both experiments, we included a greater number of social targets (e.g., targets to learn) in Experiment 1 versus Experiment 2. This is relevant because this means that the memory burden in Experiment 1 was higher than in Experiment 2. Past work in the memory domain suggests that when memory burden is higher; this can result in less detailed memory representations for studied materials (Giannakopoulos et al., 2021; Hastie & Kumar, 1979; Todorov & Uleman, 2003). Thus, it may be that the higher memory burden in Experiment 1 led participants to rely more on less detailed memory representations (i.e., impressions) for targets, which resulted in the impression memory findings in Experiment 1. In Experiment 2, where the memory burden was lower, however, participants may have been better able to rely on memory for specific details (i.e., behaviors), resulting in improved behavior memory we observed for targets associated with prosocial behaviors in Experiment 2. Interestingly, we also saw that impression memory improved for the non‐prosocial condition from Experiment 1 to Experiment 2 (which we did not predict), but this again could reflect the effects of a reduced memory burden in Experiment 2. Future work should investigate the extent that memory load affects memory for gist‐like (e.g., impressions) versus specific details (e.g., behaviors) for prosocial compared to non‐prosocial materials. Overall, given that we found better memory for prosocial information in both experiments (improved impression memory in Experiment 1; marginally improved behavior memory in Experiment 2), the results offer complementary findings in favor of a prosocial advantage in memory, which has been understudied phenomenon to date and worth future research efforts.

In Experiment 1, we used prosocial behaviors that fell into two categories: prosocial behaviors that benefitted a single individual (i.e., prosocial individual) and prosocial behaviors that benefitted more than one individual (i.e., prosocial group). Our reasoning for this was based on evolutionary theories of prosocial behavior, which argue that prosocial behaviors have been important in the development of societies (Penner et al., 2005). Based on these theories, we thought it possible that prosocial behaviors benefitting a group of people may be remembered differently as they better serve a broader social group and therefore may be more memorable. However, as we saw in Experiment 1, there was no difference between the two classes of prosocial behaviors in either of the memory measures collected, which suggests that different types of prosocial behaviors are memorable to a similar extent regardless of how many people the behavior benefits. Overall, results of the current investigation expand on the idea that prosocial behaviors are important in maintaining social groups, which is aligned with past theoretical work (Hare, 2017).

Some past work on memory advantages has taken an adaptive approach to explain the potential function of enhanced memory for certain types of information relative to others. This past work has explained memory advantages in cheater detection through an adaptive mechanism, suggesting that enhanced memory for cheaters may be useful because remembering such outcomes might help to avoid negative outcomes, such as being cheated in the future (Bell & Buchner, 2009). If such a rationale is correct, and memory for cheaters is helpful in avoiding future negative outcomes, then it may be that improved memory for prosocial behaviors may serve an analogous purpose: to remember targets who engage in prosocial behaviors because such individuals might be helpful or advantageous to interact with in the future. Said differently, if the purpose of enhanced memory for cheaters is to avoid negative future outcomes, then perhaps enhanced memory for targets engaging in prosocial behaviors may help knowing who to approach for positive outcomes that might benefit the self (i.e., being helped) in the future. Although speculative, it may be that remembering prosocial targets may guide future behaviors and induce tendencies to approach targets who might help the self. Intriguingly, recent work has started to show that memory does indeed play an essential role in social decisions, such as deciding to approach or avoid targets (Kadwe et al., 2022; Sklenar et al., 2022, 2021), and so it may be that our finding of improved memory for targets associated with prosocial behaviors may guide future behaviors to seek targets who might benefit the self. Understanding how memory may be adaptive and guide future goal‐directed behavior is an increasingly studied topic (Bell & Buchner, 2012; Frankenstein et al., 2020; Meyers et al., 2020; Nairne & Pandeirada, 2008; Nairne et al., 2007; Patel et al., 2022; Udeogu et al., 2022; Villaseñor et al., 2021), and the data from these experiments may reflect another way in which memory can be used adaptively.

Across two experiments, we found converging evidence that memory for social targets who exhibit prosocial behaviors is enhanced relative to targets who exhibit non‐prosocial behaviors as measured by both impression and behavior memory; however, there are two limitations of these data worth highlighting. First, we used impression memory to assess more gist‐like memory representations. It is worth noting, however, that impression memory may not be a “pure” measure of gist‐like representations, because it may be the case that when a participant remembers their impression for a target, they may also remember the behavior the target performed. In such instances, such memory representations would contain both gist‐like representations (accurate impression) as well as more specific details (accurate behavior). Although we found that impression memory for the prosocial behaviors was stable across both Experiment 1 and 2, it is possible that such an impure measure of gist‐like representations (e.g., impression memory) may have resulted in our inconsistent findings for the non‐prosocial targets across experiments. In the memory literature, one way to have purer estimates of gist‐like versus detailed memory representations is through use of recollection/familiarity experimental procedures (Yonelinas, 2002). In such recollection/familiarity procedures, participants are instructed to say they can “recollect” an item if they can retrieve specific details associated with that item (e.g., such as the specific behavior a social target performed), whereas if they cannot retrieve specific details but know they have encountered the item before, they are instructed to say an item is familiar. Thus, one fruitful direction for future work may be to use recollection/familiarity experimental procedures to better understand both gist‐like and detailed memory representations for targets exhibiting prosocial behaviors. Second, in both experiments, the stimuli we used for the non‐prosocial (i.e. control) condition depicted behaviors that did not contain another social target. Future work investigating the prosocial advantage in memory should do so by using a control condition that involves behaviors that do not depict prosocial behaviors, but do involve other social targets.

In this set of experiments, we investigated memory for targets showing prosocial versus non‐prosocial behaviors. We did so to better understand the extent that prosocial behaviors may be especially memorable, advancing understanding of how information about people is stored in episodic memory. Across experiments, we found complementary evidence of enhanced memory for targets associated with prosocial versus non‐prosocial behaviors as measured by impression (Experiment 1) and behavior memory (Experiment 2). Data from this investigation highlight the importance of prosociality in the social domain by demonstrating that prosocial behaviors are well‐remembered, which may be an important mechanism affecting how people navigate their social world.

## CONFLICT OF INTEREST STATEMENT

The authors have no conflicts of interest to report.

## Supporting information

Supporting Information

## Data Availability

Data will be made available upon reasonable request.

## ENDNOTES

1 To foreshadow results, we did not find any differences in memory across these two classes of prosocial behaviors, and thus this exploratory component of these stimuli will not be the main focus of this report.

2 In addition to our primary analysis of impressions, we also examined impressions formed at encoding another way by looking at the rates of positive, negative, and neutral impressions generated within each condition (e.g., the percent of prosocial individual trials that were given a positive, negative, or neutral impressions). Results of this additional analysis showed that participants formed 70% positive, 7% negative, and 23% neutral impressions for the *prosocial individual* trials. Further, participants formed 73% positive, 6% negative, and 21% neutral impressions for the *prosocial group* trials. Participants also formed 23% positive, 12% negative, and 64% neutral impressions for the *non‐prosocial* trials. These results are consistent with our primary analysis showing that participants formed more positive impressions for the prosocial trials and more negative and neutral impressions for the non‐prosocial trials. Further, it worth noting that there were few trials given negative impressions across the three conditions (prosocial individual, prosocial group, non‐prosocial). Future work might try to further investigate potential prosocial advantage in memory using procedures that induce more negative impressions.

3 As in Experiment 1, we also examined impressions formed at encoding by looking at the rates of positive, negative, and neutral impressions within both of our conditions (prosocial, non‐prosocial). Results of this additional analysis showed that participants formed 66% positive, 8% negative, and 26% neutral impressions for the *prosocial* trials at encoding. In addition, participants formed 30% positive, 17% negative, and 53% neutral impressions for the *non‐prosocial* trials. These results are consistent with our primary analysis showing that participants formed more positive impressions for the prosocial trials and more negative and neutral impressions for the non‐prosocial trials.
