# Supplementary material for: Evidence for a memory advantage for prosocial behaviors
Source: Brain Behav. 2023 Jun 8;13(7):e3096. doi: 10.1002/brb3.3096 (PMC10338796; doi:10.1002/brb3.3096)
Supplement: Supplementary file 1 — Supporting Information [file BRB3-13-e3096-s001.doc]

**Supplemental material**

To complete the norming, we recruited an independent sample of participants who rated a total of 200 behavioral sentences taken from various sources (Amato, 1990; Brown et al., 2003, 2008; Doeglas et al., 1994; Jones & Perlman, 1987; Maisel & Gable, 2009; Neff & Karney, 2005; Philippe, Rushton, et al., 1981). For each behavior, participants made four ratings. They rated the extent the sentence depicted a behavior that helped a single individual (e.g., prosocial individual behaviors) on a 1-5 rating (1 = none to 5 = a lot), as well as the extent the sentence depicted a behavior that helped a group of people (e.g., prosocial group behaviors) on a 1-5 rating (1 = none to 5 = a lot). Participants then rated how positive the behavior was (1 = not positive to 5 = very positive), as well as how negative the behavior was (1 = not negative to 5 = very negative). Based on these ratings, we then selected our final set of behavioral stimuli. Average ratings for the final set of behaviors we selected are as follows: for the “helped a single individual” question (e.g., prosocial individual behaviors), mean ratings were 4.69 for prosocial individual behaviors, 3.70 for the prosocial group behaviors, and 1.05 for the non-prosocial behaviors. For the “helped a group of people” rating (e.g., prosocial group behaviors), mean ratings were 2.56 for prosocial individual, 4.94 for the prosocial group, and 1.71 for the non-prosocial behaviors. For the positive valence rating, mean ratings were 4.84 for the prosocial individual, 4.83 for the prosocial group, and 3.71 for the non-prosocial behaviors. For the negative valence rating, mean ratings were 1.12 for the prosocial individual, 1.12 for the prosocial group, and 2.04 for the non-prosocial behaviors.
